# Putative Role of Red Wine Polyphenols against Brain Pathology in Alzheimer’s and Parkinson’s Disease

**DOI:** 10.3389/fnut.2016.00031

**Published:** 2016-08-12

**Authors:** Mario Caruana, Ruben Cauchi, Neville Vassallo

**Affiliations:** ^1^Centre for Molecular Medicine and Biobanking, University of Malta, Msida, Malta; ^2^Department of Physiology and Biochemistry, University of Malta, Msida, Malta

**Keywords:** Alzheimer’s disease, Parkinson’s disease, Mediterranean diet, red wine, polyphenols, resveratrol, neuroprotection, bioavailability

## Abstract

Alzheimer’s disease (AD) and Parkinson’s disease (PD) are the most common age-related neurodegenerative disorders and hence pose remarkable socio-economical burdens to both families and state. Although AD and PD have different clinical and neuropathological features, they share common molecular mechanisms that appear to be triggered by multi-factorial events, such as protein aggregation, mitochondrial dysfunction, oxidative stress (OS), and neuroinflammation, ultimately leading to neuronal cell death. Currently, there are no established and validated disease-modifying strategies for either AD or PD. Among the various lifestyle factors that may prevent or slow age-related neurodegenerative diseases, epidemiological studies on moderate consumption of red wine, especially as part of a holistic Mediterranean diet, have attracted increasing interest. Red wine is particularly rich in specific polyphenolic compounds that appear to affect the biological processes of AD and PD, such as quercetin, myricetin, catechins, tannins, anthocyanidins, resveratrol, and ferulic acid. Indeed, there is now a consistent body of *in vitro* and *in vivo* data on the neuroprotective effects of red wine polyphenols (RWP) showing that they do not merely possess antioxidant properties, but may additionally act upon, in a multi-target manner, the underlying key mechanisms featuring in both AD and PD. Furthermore, it is important that bioavailability issues are addressed in order for neuroprotection to be relevant in a clinical study scenario. This review summarizes the current knowledge about the major classes of RWP and places into perspective their potential to be considered as nutraceuticals to target neuropathology in AD and PD.

## Introduction

As we extend our life expectancy, population aging will inexorably increase the number of people afflicted by progressive and devastating neurodegenerative diseases, such as Alzheimer’s disease (AD) and Parkinson’s disease (PD) (1). An estimated 35 million people with AD and 10 million people with PD are affected worldwide (2, 3). Such impressive numbers place a heavy burden on the individuals concerned and inflict massive costs on society for care and treatment, at a time of difficult economic climate (4). Despite many decades of research, the available drugs for AD and PD only attenuate symptoms and have little or no effect on slowing disease progression (5, 6).

Alzheimer’s disease is by far the most common cause of dementia. It is defined by progressive loss of short- and long-term memory with a worsening cognitive deficit that leads to impaired activities of daily living (7). The consequential neuronal loss is preceded by two classical, histological lesions: (i) the extracellular accumulation of senile plaques, mainly composed of amyloid-beta (Aβ) peptide, and (ii) the formation of neurofibrillary tangles, composed of hyperphosphorylated tau proteins, mainly located in the cortex and hippocampus (8). On the other hand, PD is the most common movement disorder, its hallmark being a profound and selective loss of dopaminergic neurons in the substantia nigra *pars compacta* that manifests as motor impairment involving bradykinesia, rigidity, resting tremor, postural instability, and gait difficulty (9). This nerve-cell loss is accompanied by the presence of intraneuronal inclusions called Lewy bodies and Lewy neurites, both pathological hallmarks of PD, that consist of aggregates of a presynaptic soluble protein called α-synuclein (αS) (10).

**Figure 1:**
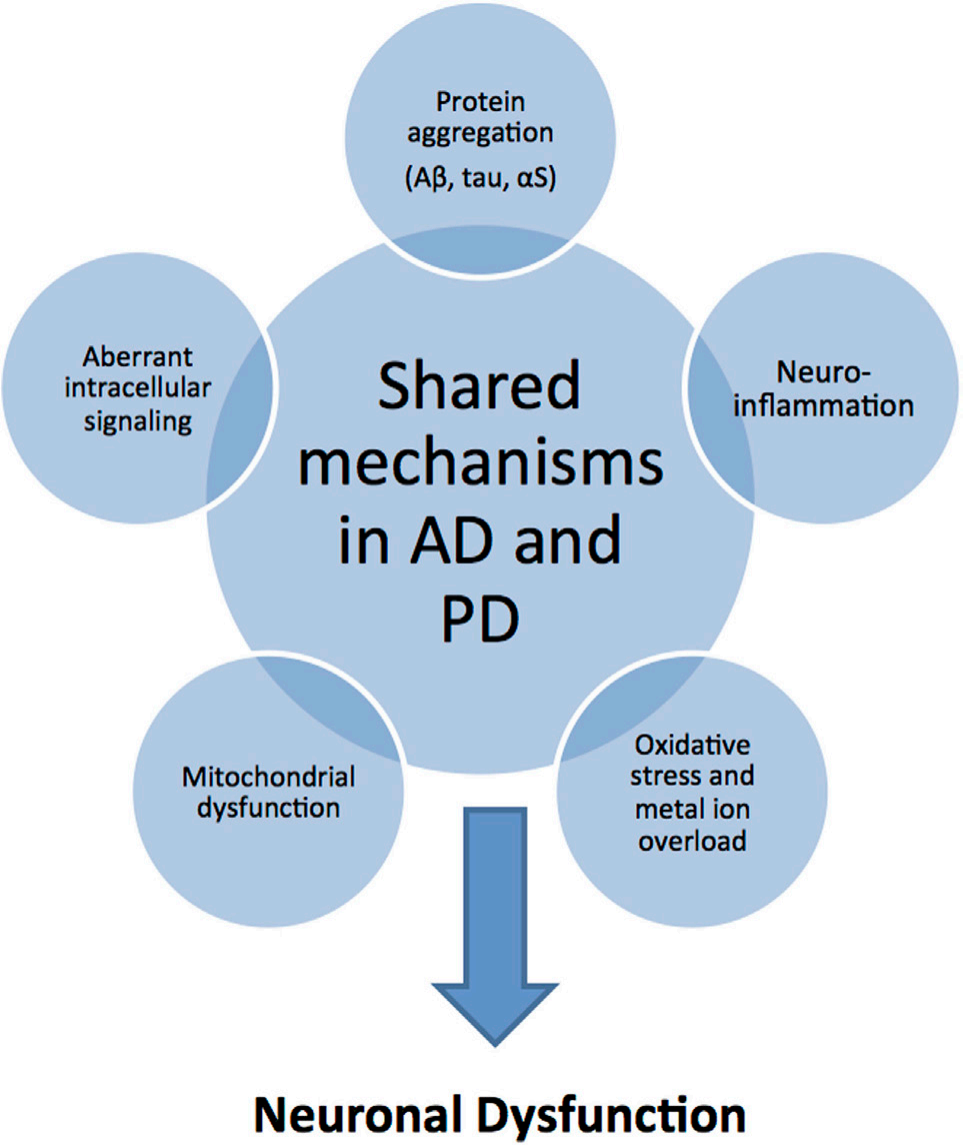
**Common pathological mechanisms shared by Alzheimer’s disease (AD) and Parkinson’s disease (PD)**. Although AD and PD have markedly different clinical and pathological features, they share common pathological mechanisms. Protein aggregation and deposition is a hallmark feature in both diseases: characteristically, amyloid plaques of amyloid-beta (Aβ) peptide and intracellular neurofibrillary tangles of tau protein in AD; Lewy bodies and Lewy neuritis of intracellular amorphous α-synuclein (αS) inclusions in PD. As a consequence or cause of protein aggregation, there is increased oxidative stress in combination with mitochondrial dysfunction related to excessive production of reactive oxygen and nitrogen species, and catalyzed by the presence of redox-active sources, such as iron overload. In addition, neuroinflammatory responses exacerbate the oxidative stress situation through the activation of aberrant cellular signaling pathways. These shared toxic mechanisms in AD and PD suggest that similar disease-modifying and therapeutic strategies may be applicable.

Although AD and PD have different clinical and pathological features, the causal mechanisms at the molecular level appear to overlap considerably (11) (Figure 1). In both neurodegenerative conditions, amyloidogenic proteins (typically, Aβ and the microtubule-associated protein tau in AD, and αS in PD) misfold and self-assemble via a nucleated-growth mechanism to form transient, low-molecular-weight soluble oligomers, later converting into β-sheet-rich protofibrils and finally stabilize as highly ordered fibrillar structures. The shared mechanism of an aberrant conversion of the native, non-toxic structure of a protein into toxic aggregates, hence, classifies both AD and PD as “protein misfolding disorders” (12). Most recent research has established that mature amyloid fibrils are not the most toxic forms of amyloidogenic proteins; rather, metastable oligomeric intermediate structures appear to be the most cytotoxic species that lead to neural dysfunction (13, 14). Several pathological events have been associated with amyloid oligomer toxicity that may lead to synaptic and neuronal dysfunction, including membrane destabilization allowing unregulated ion transport, the enhanced generation of reactive oxygen species (ROS), mitochondrial dysfunction and fragmentation, neuroinflammation, endoplasmic reticulum stress, proteasome impairment, disruption of microtubular transport, and aberrant intracellular signaling (15–17) (Figure 1). Furthermore, since amyloid oligomers are found both extracellularly and intracellularly, the capacity of small oligomers to cross cell membranes could explain the ability of protein aggregates to spread through the nervous system by prion-like spreading in AD and PD (18).

In recent years, there has been increasing supporting evidence for an association between lifestyle habits, such as diet and dietary components that might significantly delay the occurrence of AD and PD (19, 20). Particular attention has been devoted to the traditional Mediterranean diet (MeDi), which has been recognized by the United Nations Educational Scientific and Cultural Organisation as an “Intangible Cultural Heritage of Humanity.” This dietary pattern is characterized by a high consumption of plant foods (i.e., vegetables, fruits, legumes, and cereals), a high intake of olive oil as the main source of fat, a moderate intake of fish, low-to-moderate intake of dairy products, and low consumption of meat and poultry, with wine consumed in low-to-moderate amounts during meals (21). In particular, neuroprotective benefit has been attributed to a moderate consumption of wine, more specifically red wine (22). Grape is one of the richest sources of polyphenols, with red varieties containing a substantially higher polyphenolic content than white (2.5 g/L in red wine vs. 0.3 g/L in white wine) (23).

Currently, there are no established and validated strategies for the prevention or delay of onset of AD/PD, even though neurodegeneration typically develops over a long preclinical period of several decades – thus raising the possibility of a long therapeutic time window for early intervention. In this light, the possible preventive and therapeutic benefits of red wine polyphenols (RWP) are highly relevant as they display the capacity to protect neurons which goes beyond their characteristic antioxidant properties. This review attempts to highlight the diverse neuroprotective abilities of phenolic compounds found in red wine that are relevant to the common mechanisms shared between AD and PD.

## Phenolic Components in Red Wine

Natural polyphenols represent a diverse and abundant class of plant secondary metabolites found in herbal beverages and food, with no less than 8,000 phenolic structures having been identified in plants. Polyphenols are broadly classified according to the number of phenol rings and the chemical groups attached to the rings; they generally feature two aromatic rings connected via a three-carbon bridge (2-phenyl-1,4-benzopyrone) with each ring containing at least one hydroxyl group (24). Simple phenols include those compounds that have a single aromatic ring containing one or more hydroxyl groups, while the more common polyphenolic compounds are those that have multiple phenol rings within their structure. Phenols can be divided into two main groups, the flavonoids and the non-flavonoids. Flavonoid intake varies depending on the type and amount of fruit, vegetables, or beverages consumed, but averages around 1–2 g per day (25). Indicative levels of specific polyphenols in red wine are shown in Table 1.

**Table 1:** **Classification, structure, and typical amounts of the major polyphenols present in red wine, compiled from Ref. (26–29)**.

Group	Subgroup	Subclass	Main representatives	mg/L* “young”	mg/L* “aged”	Representative structure
Flavonoids	Anthoxanthins	Flavonols	Quercetin, myricetin, kaempferol, laricitrin, isorhamnetin, syringetin	100	200	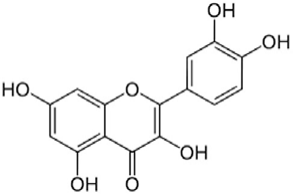 Quercitin
		Flavan-3-ols (=Flavanols)	*Monomers*: (+)-catechin, (−)-epicatechin, gallocatechin, epigallocatechin *Oligomers*: proanthocyanidins *Polymers*: condensed tannins	200 750	100 1000	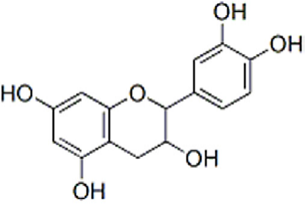 Catechin
	Anthocyanidins		Malvidin, cyanidin, peonidin, delphinidin, pelargonidin, petunidin	400	90	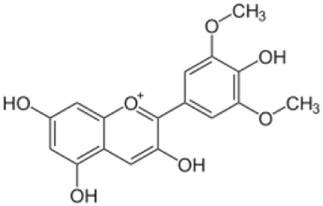 Malvidin
Non-flavonoids	Phenolic acids	Hydroxybenzoic acids	Gallic, ellagic, parahydroxybenzoic, protocatechuic, vanillic, syringic acids *Hydrolyzable tannins*: polymers of gallic and ellagic acids; castalagin, vescalagin	60 0	60 250	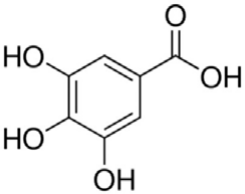 Gallic acid
		Hydroxycinnamic acids	Caffeic, coumaric, ferulic, sinapic acids	165	60	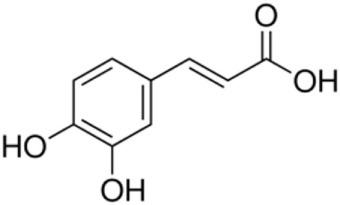 Caffeic acid
	Stilbenes		Resveratrol, piceid, astringin, piceatannol, ε-viniferin, pallidol, hopeaphenol	7	7	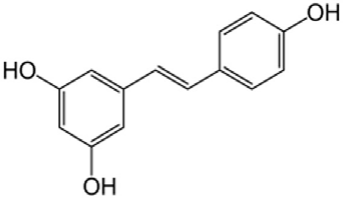 Resveratrol

*Nominal amounts for wine made from *Vitis vinifera*, the European wine grape. “*Young*” means wine less than 6 months of age and not having been aged or fermented in oak barrels. “*Aged*” implies wine about 2 years old with some oak barrel aging (or other oak contact).

**Figure 2:**
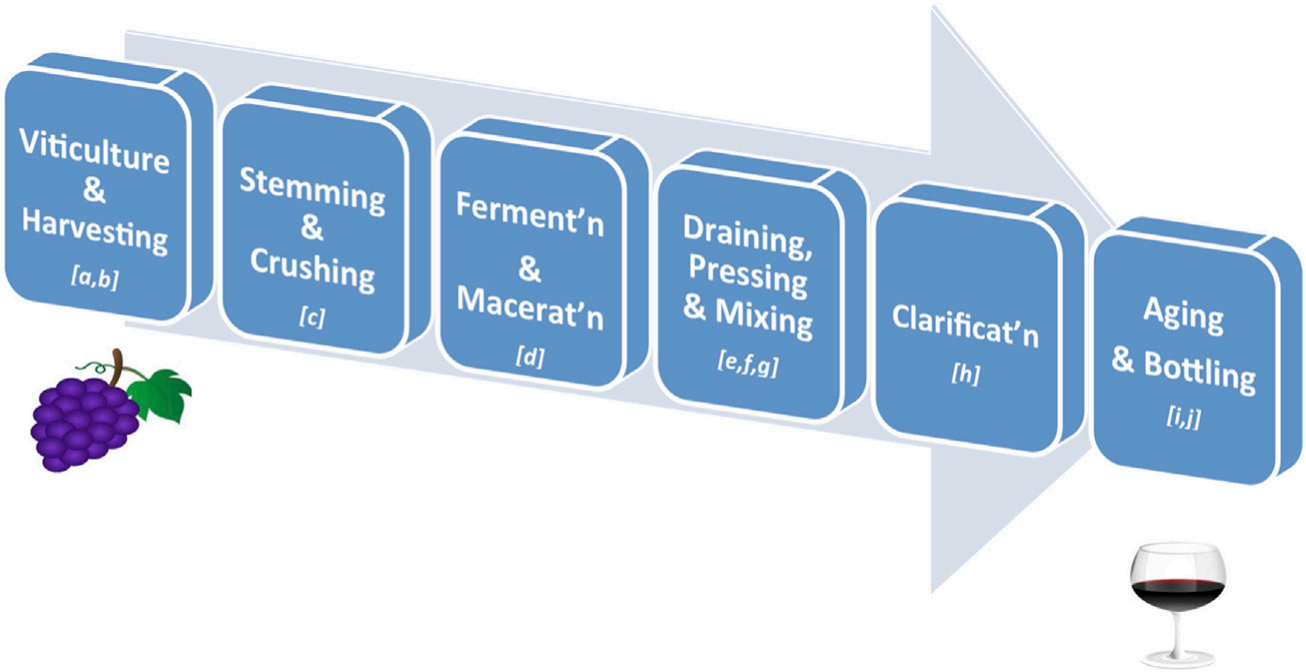
**Key stages in red wine production**. **(A)** *Viticulture*: the cultivation of grapevines; variety is affected by climate of the vineyard’s region, drainage around the vines, humidity of the region, sun exposure, and soil quality; **(B)** *Harvesting*: grapes are harvested when ripe as determined by taste, level of sugars and acid, or weather forecasts; **(C)** *Stemming and Crushing*: stemming removes the stems from the grape bunches, and crushing involves squeezing the broken grapes so that they are exposed to yeast for fermenting; **(D)** *Fermentation and Maceration*: added yeast (inoculation) will turn the sugar in wine into carbon dioxide and ethanol; this process can take from 10 to 30 days; maceration is the time given for phenolic components of the grape (such as tannins, anthocyanins) to be leached from the grape skins, seeds, and stems into the “must” (i.e., grape juice and solids); **(E)** *Draining*: the juice portion of the “must” is drained without being pressed into barrels (free-run wine); **(F)** *Pressing*: the remaining pulp (pomace – containing the skins, pulp, seeds) is pressed to squeeze out the press wine; **(G)** *Mixing*: the free-run wine and press wine, always from the same source, are mixed together in appropriate ratios to obtain the desired red wine; **(H)** *Clarification and Stabilisation*: processes by which insoluble matter suspended in the wine, such as dead yeast and grape skins, is removed before bottling; this may involve filtration, centrifugation, flotation, refrigeration, pasteurization, and/or barrel maturation and racking; **(I)** *Aging*: the clarified wine is transferred into either wooden barrels or metal vats, where the wine is allowed to further mature and develop flavors. If a winemaker chooses to age the wine in wooden casks, he will be allowing the wine to pick up tannins from the wood, adding greater depth to its flavors; **(J)** *Bottling*: done carefully so that the wine does not come in contact with air. A dose of sulfite is added to help preserve the wine and prevent unwanted fermentation in the bottle. Finer wines may be stored for several years in bottles before they are released.

Over 500 compounds have been identified in wines, with polyphenols representing the most abundant class of biologically active phytonutrients (30). Polyphenols also influence the taste, astringency, aroma, and the color of wine (31, 32). Grape phenolics are distributed in the skin, stem, leaf, and seed of the grape fruit, with 60–70% of the total being stored in the grape seed (33). Specifically, phenolic acids are largely present in the pulp, anthocyanins, and stilbenoids in the skin, while catechins, proanthocyanidins, and flavonols are found in both the skin and seeds (34). The qualitative and quantitative polyphenolic content in red wine depends on numerous factors along the wine-making process, such as (i) environmental factors in the vineyards, e.g., climate, soil, and exposure to fungal infections; (ii) grape varieties and maturity; (iii) pre-fermentative practices, such as addition of sulfur dioxide (SO_2_) and ascorbic acid (Vitamin C) before grape crushing; (iv) fermenting and aging conditions; and (v) other technological practices, such as ionic exchange, filtration, centrifugation, and cold stabilization (35–37) (Figure 2). Of particular importance is the fact that, during wine clarification, there is a decrease in the content of extractive and volatile compounds, which often translates into a significant interference in the content of polyphenols (38). Furthermore, during wine aging simple phenols present in wine are transformed into complex molecules derived from the condensation of catechins, anthocyanins, and proanthocyanidins. This results in the formation of new pigments and modification of wine color. Finally, the process of oak aging can add other phenols to the wine, most notably vanillin and hydrolyzable tannins (39). Tannins present in oaks come from lignin structures in the wood and help protect the wine from oxidation (35). Indeed, the exposure of red wine to oxygen has been established to have a strong impact on phenolic content (40). Thus, the phenolic content ultimately present in a glass of red wine is rather different from that of the non-harvested grapes. Generally, the chemical composition of the final product is much more complex than the raw material due to the formation of a variety of new polyphenolic compounds by the processes referred to above (41).

The predominant sources of phenols in red wine are flavonoids, with catechins usually the major flavanol group (26, 27, 29) (Table 1). The catechin levels in red wine are typically in the range of 20–100 mg/L, but may even rise to 1000 mg/L in old, red wines (42). The condensation of either catechin or epicatechin induces the formation of oligomers (proanthocyanidins) and polymers (condensed tannins) (28). Quercetin, myricetin, and kaempferol are found in glycoside forms and constitute the major flavonols in red wine (34). The quantity of such polyphenols varies from trace amounts up to 200 mg/L in select red wines (26). The glycosides of anthocyanidins are called anthocyanins, which typically form complex molecules with other phenolic molecules and contribute to the color and the aging of wine (43). Anthocyanins are only found in red wine, and include malvidin, cyanidin, delphinidin, peonidin, and petunidin, with malvidin being the most abundant (27).

Non-flavanoids in red wine, which include phenolic acids, hydrolyzable tannins, and stilbenes, are present in smaller quantities (Table 1). The hydroxybenzoic acids are present in their free forms and are less abundant than the hydroxycinnamates. The latter are the main class of non-flavonoid phenols in grape vine and are found as esters with tartaric acid (27, 29). The three most important hydroxycinnamates in red wine are caffeic, coumaric, and ferulic acids with typical values of 60 mg/L in aged wine (26). Hydrolyzable tannins arise during maturation and aging of wines in oak barrels where ellagic and gallic acids are the precursors (44). Castalagin and vescalagin are the main representative compounds of hydrolyzable tannins. Their levels in red wine are about 250 mg/L after the wine has aged in oak barrels for two or more years (45). Finally, stilbenes in red wine are represented by resveratrol, which can also be found in oligomeric and polymeric forms (ε-viniferins and δ-viniferins) (46, 47). The content of resveratrol in different red wines was reported to range from undetectable to 15 mg/L, with a mean value of 7 mg/L (48).

The highly complex phenolic composition of both grapes and red wine inform on the need to obtain a better polyphenolic quality control of the final red wine product. Variations in the balance and composition of RWP are translated into manifold physiological responses in biological systems. Hence, the knowledge acquired through novel technological and chemical approaches for enhancing the polyphenolic component of red wine during the wine-making process can have a positive and consistent impact on human health.

## Epidemiological Studies on Red Wine

One potential area of benefit of MeDi is that of cognitive health (49, 50). Overall, studies appear to suggest that higher adherence to the MeDi is associated with a reduced risk of developing mild cognitive impairment (MCI) and AD, and a reduced risk of progressing from MCI to AD (51–53). Analysis of pooled results from five studies, examining MeDi with a follow-up of at least 1 year, revealed that individuals in the highest MeDi score tertile had a 33% less risk of MCI or AD when compared to the lowest MeDi tertile. Nonetheless, more prospective-cohort studies and randomized controlled trials are needed to further strengthen this evidence (54). With regards to alcohol drinking, light-to-moderate drinking (one to three drinks per day) was significantly associated with a lower risk of any dementia (HR 0.58; 95% CI, 0.38–0.90) and vascular dementia (HR 0.29; 95% CI, 0.09–0.93) in individuals aged 55 years and over (55). In a similar vein, there were lowest odds for dementia among older adults whose weekly alcohol consumption was one to six drinks every week (OR 0.46; 95% CI, 0.27–0.77), regardless of the type of beverage consumed, when compared to abstainers (56). Specifically addressing the role of red wine, an inverse association between moderate wine drinking and incident dementia in the elderly has long been proposed (57). This has been supported by a cohort study (*Copenhagen City Heart Study*) among individuals aged 65 years and older, in which it was found that monthly or weekly intake of wine, but not other alcoholic drinks, was associated with a lower risk of dementia including AD; suggesting that certain substances in wine may reduce the occurrence of dementia (58). An oft-cited study in which the volume of red wine intake has been recorded is a 3-year prospective study performed in a cohort of 3,777 community residents aged 65 and over. Moderate drinking of three to four glasses per day (or 250–500 mL/day) of red wine was associated with a fourfold diminished risk of AD and incident dementia when compared to those who drank less, or did not drink at all (57). Despite an overall positive association of red wine consumption with better cognitive health, whether people should start drinking or increase wine consumption to avoid dementia is still debatable. Larger, prospective-cohort studies with a longer follow-up and further randomized controlled trials are warranted.

Concerning PD, a habitual dietary intake of flavonoids has been found to be protective against PD risk. More specifically, male participants in the highest quintile of total flavonoid intake had a 40% lower PD risk than those in the lowest quintile (HR 0.6; 95% CI, 0.43–0.83). Intakes of anthocyanins from flavonoid-rich foods, including red wine, were especially associated with the lower PD risk (59). Considering MeDi and Parkinson’s risk, a reduced odds for PD age at onset (OR 0.86; 95% CI, 0.77–0.97) was associated with a higher MeDi score; while lower MeDi diet adherence was associated with earlier PD (60). On the other hand, most epidemiological studies to date do not support an association between alcohol or wine consumption and risk of PD (61, 62).

To conclude, it is still premature, on the currently available epidemiological data, to be able to advise all elderly people to drink wine regularly for the prevention of age-related neurodegeneration. Moreover, any public health message concerning regular wine intake also has to address the risk-to-benefit ratio associated with excessive ethanol consumption present in wine, which may lead to adverse outcomes of intoxication, hypertension, cardiomyopathy, stroke, and oral cancer (63, 64). Ultimately, the epidemiological analysis of the relations between wine consumption and mental decline is complex, and it is highly unlikely that a single component plays a major role (65). General limitations in epidemiological studies assessing the role of red wine and red wine components in neurodegenerative disease include: (i) the assumption that wine intake will remain unchanged over the time period of the study; (ii) confounders such as other components of the diet (e.g., tea) which may contribute to the total amount of polyphenols consumed; (iii) variability in the content and composition profile of RWP (as discussed above); and (iv) the lack of reliable diagnostic tools for AD and PD (66). Nevertheless, although challenging, epidemiological research is indispensable to support the need for intervention trials designed to test the epidemiological associations between red wine and age-related neurodegeneration. Moreover, a better scientific understanding of the role of specific dietary polyphenolic components in red wine that are capable of exerting beneficial disease-modifying activities, and their mechanisms of action, may point the way toward therapeutic application of select red wine phenols in AD and PD.

## Neuroprotective Effects of Red Wine Polyphenols in AD and PD

Much effort has been undertaken in the way of understanding the neuroprotective effects of polyphenols, using both *in vitro* and *in vivo* models (67). Broadly speaking, the molecular mechanisms of their neuroprotective actions can be classified as: (i) anti-inflammatory activities and antioxidant capacity, including free radical scavenging and metal chelation; (ii) modulation of cell signaling pathways; and (iii) anti-amyloid action through direct binding with specific amyloidogenic proteins such as Aβ, αS, and tau. Such a wide mode of action highlights a key aspect that has repeatedly emerged from studies on natural polyphenols, including wine polyphenols – namely that they exhibit a remarkable ability to simultaneously and synergistically modulate multiple molecular targets, suggesting a greater potential for therapeutic efficacy in the complex pathogenesis of AD and PD (22, 68). Thus, in this section, we summarize *in vitro* and *in vivo* neuroprotective effects of RWP in relation to the shared pathological features involved in AD and PD. Experimental findings on neuroprotective mechanisms will later be discussed in light of recent bioavailability and clinical studies aimed at therapeutic use of red wine polyphenols.

### Red Wine Polyphenols Reduce Oxidative Stress through Direct Antioxidant and Iron-Chelating Activity

The central nervous system is highly susceptible to oxidative stress (OS), mainly due to its high oxygen consumption and metabolic activity (69). Other reasons for the selective neuronal variability to OS include the presence of elevated amounts of redox-active metals, such as zinc, copper, and iron, and the enrichment of neuronal membranes with long-chain polyunsaturated fatty acids which are extremely sensitive to oxidation (69, 70). Because of this, OS has been majorly implicated in the pathogenesis of neurodegenerative diseases and, hence, direct antioxidant and metal-complexing properties of RWP may be of significance (71).

The oft-described antioxidant activity of red wine is explained mostly by its polyphenol content. A plethora of *in vitro* studies have described the potent free radical scavenging effects of RWP, including direct scavenging of reactive oxygen and nitrogen species, such as peroxides, superoxide, the hydroxyl radical, and the peroxynitrite anion, as well as sequestering of highly redox-active metal ions (72). For instance, major antioxidant polyphenols found in Merlon wine extract included quercetin, catechin, epicatechin, tyrosol, gallic acid, and procyanidins, with quercetin and procyanidins being the most active antioxidants. Pretreatment of neuronal and astrocytic cell lines under OS conditions with these polyphenolic compounds suppressed ROS production and significantly improved cell viability (73, 74). Another important wine polyphenol, resveratrol, was cytoprotective in human neuroblastoma cells exposed to Aβ or to Aβ-metal complexes via its scavenging properties (75). In a similar manner, anthocyanins, ferulic acid and other hydroxycinnamic acids, variously protected against protein oxidation and lipid peroxidation in solution, in neuronal cell lines, and in synaptosomal systems exposed to reactive nitrosative species, both at membrane and cytosolic levels (76–78). Lipid nanoparticles entrapping ferulic acid reduced lipid peroxidation of rat brain microsomes; such new formulations could promote uptake of ferulic acid by cells because of their lipid-based structure (79). Pretreatment of cortical neurons with quercetin, caffeic acid, and metabolic derivatives of catechin and epicatechin protected against injury by OS-induced, highly neurotoxic catecholamine-quinones and cysteinyl-catecholamine conjugates (e.g., 2-S- and 5-S-cysteinyl-dopamine) that are thought to be relevant to the etiology of PD (80). A novel approach to antioxidant therapy includes the development of protective compounds targeted to mitochondria. Apart from representing the major site of superoxide-generation within neurons, mitochondria are also a major target for the oxidative action of ROS. Consistent evidence suggests that mitochondrial dysfunction is central to the early events in the pathogenesis of AD and PD (81, 82). In PD, therapy with L-DOPA itself may give rise to additional oxidative/nitrosative stress targeting mitochondria in dopaminergic neurons, as evidenced in rats. In the *substantia nigra* and striatum of rats administered L-DOPA, there was an increase in oxidized glutathione and inducible nitric oxide synthase (iNOS) upregulation that was accompanied by induction of the cytoprotective heat shock protein (Hsp) 70 and the mitochondrial chaperone Hsp60 aimed at re-establishing mitochondrial homeostasis (83). Several individual polyphenols present in wine have been demonstrated in *in vitro* studies to exert promising mitochondrial protection. These include anthocyanidins and proanthocyanidins, quercetin, and resveratrol [reviewed in Fernandez-Moriano et al. (84)].

It should be emphasized at this point that when considering the final antioxidant effect of red wine, possible synergistic/additive/antagonistic effects among the various polyphenol compounds in the mixture should be taken into account. In fact, a study on the interaction of three RWP – quercetin, resveratrol, and caffeic acid – in combination revealed markedly different antioxidant/scavenging potencies of the compounds when compared to the activity of individual polyphenols alone (85).

*In vitro* findings have driven further research on the antioxidant effects of RWP in animal models. Resveratrol administration, both intravenously or as a dietary supplement, significantly protected mice from motor co-ordination impairment induced by 1-methyl-4-phenyl-1,2,3,6-tetrahydropyridine (MPTP), a parkinsonian neurotoxin. Furthermore, resveratrol protected against hydroxyl radical overloading of striatal neurons and prevented depletion of striatal dopamine (86, 87). In a rat model of PD induced by intrastriatal injection of another OS-generating neurotoxin, 6-hydroxydopamine (6-OHDA), resveratrol upregulated antioxidant enzyme activity and improved antioxidant status while lowering dopamine loss (88). *In vivo* supplementation of grape seed extract (GSE) enriched in proanthocyanidins to aged rats (100 mg/kg body weight for 30 days) inhibited the accumulation of oxidative DNA damage and normalized lipid peroxidation and antioxidant defenses (89, 90). In humans, drinking 300 ml of red wine every day for a week (mild-to-moderate consumption) improved the profile of antioxidant enzyme expression and activity in blood (91).

Generally, *in vivo* work has shown that although the antioxidant properties of wine have been largely attributed to the ROS-scavenging ability of constituent phenolics, direct scavenging is limited *in situ* in the brain and, therefore, unlikely to be the main mechanism of action. Rather, other indirect mechanisms for their neuroprotective effects are most probably exerted through influences on the intracellular redox status, such as (i) inhibition of redox-sensitive transcription factors, e.g., nuclear factor-κB (Nf2) and activator protein-1 (AP-1); (ii) upregulation of antioxidant enzymes, e.g., glutathione S-transferases and superoxide dismutases; and (iii) inhibition of pro-oxidant enzymes, e.g., nitric oxide synthase, xanthine oxidase, cyclooxygenases, and lipoxygenases (92).

### Red Wine Polyphenols Modulate Signaling Pathways

It has become evident that RWP and their corresponding *in vivo* metabolites elicit their neuroprotective effects not by simply acting as antioxidants, but rather by interacting with various signaling cascades involved in adaptive stress responses (93). Selective inhibitory or stimulatory actions of RWP on neuronal and glial kinase signaling cascades have been studied, including (i) phosphoinositide 3-kinase (PI3K)/protein kinase B (Akt); (ii) mitogen-activated protein kinase (MAPK) and extracellular signal-regulated protein kinase (ERK1/2); (iii) nuclear factor erythroid 2-related factor 2 (Nrf2); and (iv) nuclear factor kappa B (NFkB) (93). Inhibition or stimulation of these pathways by RWP is likely to profoundly affect cellular function by altering the phosphorylation state of target molecules and/or by modulating gene expression (94). Such actions will be highlighted in relation to the pathogenesis of AD and PD (Table 2).

**Table 2:** **Neuroprotective signal transduction by major red wine polyphenols and their metabolites**.

RWP	Stimulation (+) inhibition (−)	Signaling pathway/s	Neuroprotection	Reference
Resveratrol, (−)epicatechin	+	Nrf2/HO-1/ARE	Attenuate OS and neuroinflammation through the expression of protective enzymes and scavengers	Shah et al. (95); Ren et al. (96)
Resveratrol, ferulic acid, epicatechin, quercetin, *O-*methylated quercetin	+ −	MAPK/ERK1/2 MAPK/JNK	Neuronal growth factor-induced mitogenesis, differentiation; anti- apoptotic; enhanced neuronal survival and plasticity	Dasgupta and Milbrandt (97); Zhang et al. (98); Zeni et al. (99); Schroeter et al. (100); Schroeter et al. (101); Spencer et al. (102)
Kaempferol, resveratrol, pterostilbene, quercetin	−	NF-κB	Inhibit neuroinflammation; suppress oxidative damage	Capiralla et al. (103); Zhang et al. (104); Jin et al. (105); Chang et al. (106)
Resveratrol, quercetin, *O-*methylated quercetin	−	PI3K/Akt	Increased neuronal survival and plasticity; inhibition of mitochondrial-mediated apoptosis	Spencer et al. (102); Simao et al. (107)

*Activation of signaling pathways is shown as (+), while downregulation of signaling pathways is shown as (−)*.

*ARE, antioxidant response element; Akt, protein kinase B (PKB); ERK, extracellular signal-regulated protein kinase; HO-1, Heme oxygenase-1; JNK, c-Jun N-terminal kinase; MAPK, mitogen-activated protein kinase; NFκB, nuclear factor kappa B; Nrf2, nuclear factor erythroid 2-related factor 2; PI3K, phosphatidylinositol-3 kinase*.

The best-characterized MAPK pathways are the mitogenic ERK and the stress activated c-Jun N-terminal kinase (JNK) signaling pathways (94). The potential modulation of MAPK signaling by RWP is significant, as ERK1/2 and JNK are involved in neuronal growth factor-induced mitogenesis, differentiation, apoptosis, and neuronal plasticity (108). Investigations have indicated that individual RWP and/or their metabolites may interact selectively within the MAPK signaling pathways (109). For example, through the activation of ERK1/2, resveratrol and ferulic acid significantly enhance mammalian neurotrophins, such as nerve growth factor (NGF) and brain-derived neurotrophic factor (BDNF) in neuronal cell lines (97–99). Modulation of neurotrophin signaling is crucial to support neuronal survival and maintain synaptic plasticity, hence, might provide a therapeutic strategy in AD and PD (110). Interestingly, the ability of resveratrol to protect hippocampal cells against Aβ-induced toxicity correlated strongly with its affinity to “receptor” binding sites at the level of the cellular plasmalemma in rat brain (111). Epicatechin and one of its major *in vivo* metabolites, 3′*-O*–methyl-(−)-epicatechin, stimulated phosphorylation of ERK1/2 at physiologically relevant concentrations thereby protecting neurons against OS-induced apoptosis via a mechanism involving the suppression of JNK (100, 101). On the other hand, neither quercetin nor its *O-*methylated metabolites had a measurable effect on JNK phosphorylation (102).

In addition to MAPK pathways, RWP have been identified to modulate signaling through Akt, one of the main downstream effectors of the PI3K pathway, and a pivotal kinase in controlling neuronal survival and apoptosis. For instance, resveratrol given by intraperitoneal administration to rats has been reported to protect against ischemic neuronal cell death in the CA1 hippocampus, via downregulation of glycogen synthase kinase 3 (GSK-3β) and cAMP response element-binding protein (CREB) protein expression, through activation of PI3K/Akt signaling (107). On the other hand, quercetin is neurotoxic in primary cortical neurons by potent inhibition of survival signaling through PI3K/Akt (102). Indeed, it should be noted that interactions between intracellular signaling cascades and RWP could have unpredictable outcomes depending on the polyphenol combination, concentrations used, and cell type.

The MAPK, ERK, and PI3K/Akt pathways can all activate nuclear factor E2-related factor 2 (Nrf2) signaling, which pathway coordinates the enhanced expression of a large number of prosurvival genes that allows neurons to respond to various conditions of stress, including OS (112). In particular, the Nrf2-antioxidant response element (ARE) pathway leads to the downstream expression of several enzymes with antioxidant and detoxification capacities. Not surprisingly, therefore, given the importance of OS and neuroinflammation, plenty of evidence highlights the neuroprotective roles of the Nrf2–ARE pathway in AD and PD (113). Many studies clearly demonstrate that dietary polyphenols strongly induce the Nrf2–ARE pathway in neurons and astrocytes, and this mechanism could prevent cognitive decline and neurodegeneration (114). One of the enzymes that is under the control of ARE in brain cells is heme oxygenase 1 (HO-1), an enzyme degrading heme to carbon monoxide, free iron, and biliverdin (115). Thus, among wine-phenolic components, ferulic acid exerted neurprotection by increasing HO-1 activity in a human neuroblastoma cell line (116), while resveratrol and epicatechin pretreatment protected against focal cerebral ischemia in rats and mice, respectively, by upregulating expression of Nrf2 and activation of HO-1 to ameliorate oxidative damage (95, 96). In a rat model of AD induced by inoculation of the Aβ peptide, resveratrol administration increased expression of HO-1 and reduced lipid peroxidation, thus improving spatial memory (117).

Another cardinal transcriptional regulator of inflammation and apoptosis is NF-κB; upregulation of NF-κB leads to increased inflammatory signaling (118). Chronic neuroinflammatory processes mediated by NF-κB significantly contribute to the initiation and progression of neuronal damage observed in AD and PD, thus making selective targeting of NF-κB an attractive therapeutic strategy (119, 120). Polyphenols, including those found in red wine, readily attenuate NF-κB activation by targeting multiple inflammatory cascades, such as MAPK/ERK1/2, PI3K/Akt/JNK, and others (121, 122). Considering resveratrol, studies in cell cultures showed that by preferentialling inhibiting NF-κB activation, it prevented the pro-inflammatory effect of fibrillar Aβ peptides on microglia. Consistent with this effect, orally administered resveratrol lowered microglial activation in a mouse model of cerebral amyloid deposition (103). In a PD model of rat primary midbrain neuron–glia cultures, resveratrol also exhibited neuroprotective effects through inhibition of microglial activation and subsequent decrease in pro-inflammatory factor release. These effects were related to downregulation of MAPK and NF-κB pathways in microglia (123). *In vivo*, resveratrol given orally for 10 weeks reduced neural inflammation in a 6-OHDA-induced PD in rats (105). In a similar PD model in zebrafish, another major polyphenol found in red wine, quercetin, prevented 6-OHDA-stimulated dopaminergic neuron loss by reduction of pro-inflammatory gene expression (104). Interestingly, the oral administration of an extract containing resveratrol to healthy subjects for 6 weeks was reported to have a comprehensive suppressive effect on OS and inflammatory indices with a decrease in NF-κB binding (124). Recently, pterostilbene, a derivative of resveratrol which is more lipophilic, exhibited stronger modulation of neuroinflammation than the parent compound in a mouse model of accelerated aging (106).

As such, therefore, phenolic constituents of red wine represent potent small-molecules capable of countering OS and neuroinflammation in neurodegenerative disease. Such regulation appears to be mediated by attenuation of microglial activation and associated actions on diverse intracellular signaling pathways, including the MAPK cascade and NF-κB pathway. Perhaps further work should be conducted to elucidate the consequences of the interactions or the synergistic effects between different RWP on their myriad intracellular targets (121).

### Red Wine Polyphenols Antagonize Formation of Toxic Amyloid Aggregates

As previously mentioned, another pathological hallmark shared by both AD and PD is the misfolding and aberrant self-association of amyloidogenic proteins (e.g., Aβ, αS, and tau) into neurotoxic higher-order aggregates (12). Especially toxic are the lower molecular weight, soluble protein oligomers that play a key role in the functional impairment and death of neurons (125). Lately, further overlap between AD and PD at the molecular level is being revealed by a direct interaction of tau with αS; the two amyloidogenic proteins catalyze the polymerization of each other, triggering the formation of toxic tau/αS co-oligomers, which eventually leads to deposition of the co-aggregates (126). These common aggregation processes imply that preventing the accumulation of toxic oligomeric species in the brain might provide a useful therapeutic approach (127). In fact, select polyphenols found in red wine possess robust anti-amyloidogenic properties (128). Small-molecule polyphenolic compounds may alter the aggregation pathways by a number of different mechanisms: (i) stabilize the “benign” native form of amyloidogenic proteins; (ii) block the early assembly processes in the aggregation pathway, preventing the formation of toxic oligomers; (iii) inhibit fibril growth and extension; (iv) disassemble preformed fibrils into non-toxic confomers; and (v) inhibit amyloid–membrane interactions (129–132).

In a study carried out on 39 different flavonoids, wine-related polyphenolic compounds, especially quercetin, exhibited strong inhibitory effects against Aβ fibril formation *in vitro* (133). On the other hand, ferulic acid did not prevent fibril formation, although it modified the length of Aβ fibrils and still protected against Aβ toxicity in transgenic *Caenorhabditis elegans* (134). Quercetin also displayed fibril-destabilizing effects on preformed fibrillar Aβ and reversed Aβ-induced neurotoxicity in a cell system overexpressing APP Swedish mutation (APPswe), which is associated with early-onset familial AD (135). The polyphenol metabolite quercetin-3-*O-*glucuronide is also capable of interfering with the formation of neurotoxic oligomeric Aβ species. Interestingly, this quercetin metabolite was found to accumulate in rat brain following oral dosage with Cabernet Sauvignon red wine, and improved AD-type deficits in long-term potentiation by promoting neuroplasticity processes (136). In line with these findings, it has been demonstrated that consumption of wine obtained from Cabernet Sauvignon grapes by transgenic Tg2576 mice (which model AD-type amyloid-β neuropathology) significantly suppressed AD phenotypes by preventing toxic Aβ peptide generation (137). Another wine, muscadine wine, suppressed memory deterioration in the same transgenic AD mice by interfering with the oligomerization process of Aβ (138). The mechanism of action of resveratrol involves direct binding to Aβ, interference with Aβ aggregation and formation of “off-pathway” Aβ oligomers that have reduced cytotoxicity (139). Of relevance to the *in vivo* situation, resveratrol and its derivatives identified in wine, such as piceid and ε-viniferin glucoside (a resveratrol dimer) strongly inhibited fibrillization of Aβ peptide and protected PC12 cells against Aβ-induced toxicity (140). Another wine-related polyphenol, ellagic acid, attenuated Aβ-induced neurotoxicity in cell culture by accelerating fibril formation and simultaneously causing a significant reduction in toxic intermediate oligomeric species (141).

A number of studies to investigate the protective effects of red wine have made use of a commercially available grape seed polyphenolic extract (GSPE) that is rich in gallic acid, catechins, and proanthocyanidins. GSPE significantly inhibited Aβ aggregation *in vitro* and when administered orally in a mouse model of AD it reduced Aβ plaques and attenuated AD-type cognitive deterioration (142, 143). From a structural-activity analysis of GSPE compounds, it was concluded that the most effective Aβ aggregation inhibitors were in fact the polyphenol oligomers present in the GSPE mixture (144). Moreover, anti-aggregation properties of GSPE were observed in the inhibition of tau peptide aggregation, as well as the dissociation of preformed tau aggregates, possibly through non-covalent interactions of GSPE-derived polyphenols with tau residues (145). GSPE was also effective in the *in vivo* scenario, such that GSPE delivered through drinking water significantly reduced levels of toxic hyperphosporylated tau and improved motor phenotype of transgenic mice expressing a human tau protein containing the P301L mutation (146). Another extract related to red wine components, known as BDPP (Bioactive Dietary Polyphenol Preparation – consisting of a mixture of Concord grape juice, GSE, and resveratrol) mitigated amyloid load, loss of synaptic plasticity, and cognitive impairment in mouse models of AD. Significantly, as has been observed elsewhere in this review, combinatory treatment with the extract preparation was much more effective than treatment with the individual polyphenols alone (68). Fewer studies have made use of wine extracts, or a combination of wine-related constituents, in relation to models of PD. One example is a grape extract (Regrapex-R^®^) prepared from whole grape (*Vitis vinifera*) fed to transgenic Drosophila expressing human αS (a fly model of PD), which resulted in a significant improvement in climbing ability compared to controls (147). Aggregation of αS in the brain has been implicated as a critical step in the development of PD and related synucleinopathies. Therefore, components of red wine, which hinder αS aggregation, may prove effective as disease-modifying drugs in PD. Wine-related polyphenols that reportedly inhibited the formation of fibrillar αS, and destabilized preformed fibrillar αS in a dose-dependent manner include: tannic acid = myricetin > kaempferol = ferulic acid > catechin = epicatechin (148). The two most potent RWP, tannic acid and myricetin, also displayed strong inhibition of αS oligomer formation and disaggregated preformed oligomers *in vitro* (149). A detailed investigation revealed that myricetin inhibited αS oligomerization by directly binding to the N-terminal region of αS (150). More recently, quercetin was also shown to covalently bind αS, with the increased hydrophilicity of the covalently modified αS accounting for the inhibition of aggregation (151).

## Metabolism and Bioavailability of Red Wine Polyphenols

**Figure 3:**
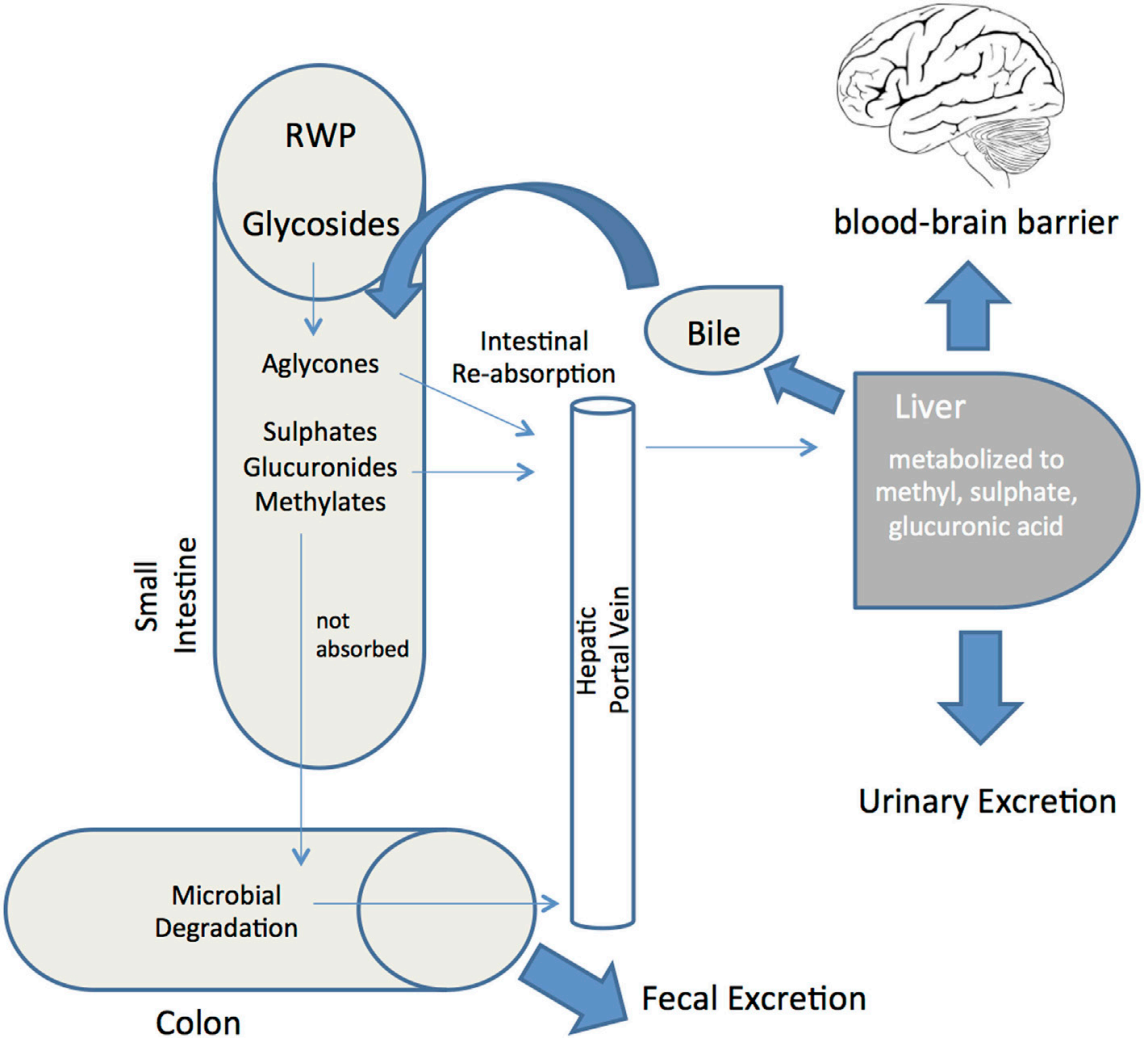
**Red wine polyphenols metabolism and absorption in the human digestive system**. Schematic depiction of the metabolic fate of red wine polyphenols (RWP) after ingestion into the gastrointestinal tract. Following ingestion, enzymes hydrolyze glycosylated RWP and then aglycones enter epithelial cells by passive diffusion. RWP that are not absorbed reach the colon and proceed to microbial degradation before colonic absorption. Once a final derivative or aglycon has been absorbed it undergoes phase I/II metabolism at enterocyte level to produce sulfates, glucuronides and methylates. These metabolites then enter the blood stream by the portal vein, reaching the liver, where they may be subjected to more phase II metabolism, thence becoming conjugated and transported to the bloodstream again until they are secreted in urine. Some of the liver conjugates are excreted as bile components back into the small intestine (enterohepatic circulation). Unabsorbed metabolites are eliminated via feces. Bioavailability is defined as the percentage of a RWP that is absorbed into the bloodstream and available to exert its effect at the target tissue (brain through the blood–brain barrier).

Whether the multiple biological activities of RWP described above translate into actual pharmacological effects *in vivo* depends very much on their bioaccessibility and systemic bioavailability. Bioaccessibility is defined as the amount of an ingested compound that is present in the gut as a consequence of release of this constituent from the solid food matrix, whereas bioavailability is the proportion that is absorbed, metabolized and available to exert its biological effects at the target tissue (42, 152). Although the typical modifications that occur during and after absorption of several common polyphenols are reasonably well understood, the bioavailability of polyphenols is determined by interplay of absorptive and metabolic pathways (Figure 3). Indeed, unraveling the bioavailability of natural polyphenols is more challenging than with synthetic compounds (153), not least because resident gut microbiota generate secondary metabolites (154). This typically involves deglycosylation, followed by breakdown of ring structures to produce phenolic acids and aldehydes. After absorption, metabolites can be glucuronidated, sulfated, and/or methylated and are detected in bloodstream, urine, and fecal samples (155, 156).

Notwithstanding the complex metabolic fate of polyphenols, it is well acknowledged that RWP may delay pathological processes leading to AD/PD by accumulating in the brain of mouse and rodent models. For example, major GSPE components, such as gallic acid, catechin, epicatechin, and their metabolically derivatized forms, were identified in the plasma of rats following acute gavage with GSPE; however, only repeated dosing resulted in brain deposition of metabolites (157). An epicatechin metabolite, 3′-*O*-methyl-epicatechin-5-*O*-β-glucuronide accumulated in the hippocampus after oral administration of GSPE, where it promoted basal mechanisms related to learning and memory at physiologically relevant concentrations (158). Rapid absorption of catechin and epicatechin into plasma was shown in orally administered rats, with plasma concentrations peaking at 2–3 h after ingestion (159). In human volunteers, metabolites of catechin and epicatechin, mainly glucuronides and methyl glucuronides, were detected in plasma only after regular red wine drinking (~375 ml of red wine daily for 2 weeks) (160). Long-term feeding of rats with quercetin diet also resulted in deposition of quercetin and its metabolites in rat brain (161). Therefore, it appears that the maintenance of a high plasma concentration of polyphenols and their metabolites in living organisms requires repeated ingestion over time.

Oral bioavailability data on resveratrol indicate extensive metabolism and generally poor bioavailability (162). Nevertheless, a growing number of studies describe bioactivity of resveratrol *in vivo*, implying that resveratrol does exert protective effects in animal models of disease (163, 164). In this respect, it is believed that accumulation of biologically active resveratrol metabolites may mediate the neuroprotective effects. In humans, too, resveratrol glucuronides, rather than free resveratrol, was detected in plasma after moderate consumption of red wine (165). Such a scenario would explain why dietary supplementation with clinically feasible doses of resveratrol reduced beta-amyloid plaque formation and OS in a transgenic mouse model of AD, despite no detection of resveratrol in the brain (166). Two months of dietary supplementation with pterostilbene, a resveratrol analog, also improved AD pathology and cognition in a transgenic mouse model of aging (106). In light of bioavailability studies, further exploration of the potential beneficial roles of RWP in AD and PD models should include the use of specific metabolites that with repeated dosing are known to accumulate in the brain. This in turn signifies that the plasma pharmacokinetics and tissue distribution of polyphenols and their metabolites have to be delineated more extensively, using advanced methods such as high-performance liquid chromatography (HPLC), mass spectrometry (MS), and liquid chromatography (LC) (167). Improving the bioavailability of RWP with therapeutic implications for AD and PD entails ensuring efficient transport across the blood–brain barrier (BBB) and delivery to the brain (168). Many recent advances report that RWP metabolites are able to cross the BBB and accumulate in the brain at pharmacologically relevant nanomolar or micromolar concentrations (136, 157, 169). However, the interaction of polyphenolic metabolites with the BBB has not been sufficiently investigated. Polyphenols penetration through the BBB is dependent on the degree of lipophilicity of each compound, with less polar metabolites (e.g., *O*-methylated derivatives) capable of greater brain uptake in comparison to the more polar metabolites (e.g., sulfated and glucuronidated derivatives) (170). Stereoactive interaction with specific efflux transporters expressed on endothelial cells of the BBB has been observed, and is another factor determining brain permeability (171). An exciting new development in surmounting the BBB obstacle and ensuring better polyphenol delivery into the brain is represented by new delivery systems, such as those based upon lipid-core nanoparticles (entrapment of polyphenols in lipid vesicles) (172). Thus, resveratrol concentration in brain tissue was significantly increased by intraperitoneal administration of nanoencapsulated resveratrol, and the polyphenol was able to rescue rats from the damaging effects of Aβ injection much better than free resveratrol (173, 174). Further knowledge gleaned from much-needed research on the bioavailability of RWP, and on delivery of metabolites across the BBB, will be especially useful for conducting better-designed human clinical interventional trials.

## Clinical Interventional Studies

Although much is known about the potential antioxidant and neuroprotective roles of RWP, few clinical trials have been conducted to quantify therapeutic benefits in AD and PD, with conflicting conclusions at best (175). Published clinical trials of resveratrol were largely focused on characterizing its pharmacokinetics and metabolism, or improve specific parameters, such as memory or physical performance in adults (e.g., ClinicalTrials.gov: NCT01126229). In recent studies, resveratrol was found to be safe and reasonably well-tolerated at doses of up to 5 g/day in humans (176). Single oral doses (250 and 500 mg) of resveratrol given to healthy adults improved cerebral blood flow, but not short-term cognitive performance (177). Three clinical studies have been carried out that explored the benefits of resveratrol for treating individuals having AD or MCI, a clinical condition that is often a precursor to Alzheimer’s dementia. Between 2008 and 2010, resveratrol supplementation was investigated in a randomized placebo-controlled phase III trial involving 27 mild-to-moderate AD individuals, with the primary endpoint of assessing Alzheimer’s Disease Assessment Scale-cognitive subscale (ADAS-cog scores) (clinicaltrials.gov NCT00678431). However, results from this study have not yet been published. In 2013 and 2014, a multi-center phase II trial of resveratrol was conducted in 119 individuals with mild-to-moderate AD. Participants were randomized to either placebo or resveratrol (500–2,000 mg daily). Resveratrol was safe and well-tolerated and, even though only 1% of resveratrol reached central nervous system, AD biomarker changes were associated with resveratrol treatment. These include a significantly less pronounced decline in cerebrospinal fluid and plasma amyloid-beta levels (6% vs. 20%, resveratrol-treated group vs. placebo, respectively), suggesting that resveratrol had indeed engaged its target in the brain (178). Another ongoing, multi-interventional phase II clinical trial involves 330 subjects with MCI who were given resveratrol supplementation as one of six different interventions or placebo, with the ADAS-cog score as the primary outcome (clinicaltrials.gov NCT01219244).

In relation to PD, a large prospective study carried out over two decades involving almost 130,000 individuals showed that the habitual intake of flavonoid-rich food and beverages, including red wine, was significantly related to a reduced risk of developing the disease (40% lower PD risk for participants in the highest quintile compared to those in the lowest quintile) (59). Nevertheless, the authors caution that the results must be confirmed by other large prospective studies carried out on populations with a wider heterogeneity in dietary flavonoid intake.

Rounding up, more convincing large-scale clinical trials utilizing RWP are needed, together with suitable biomarkers, to objectively assess a risk reduction of AD and PD. Clinical interventional trials must be prioritized to support extensive evidence derived from *in vitro* and *in vivo* studies.

## Conclusion and Future Directions

An increasingly accepted notion is that wine-related compounds exert neuroprotective and neurorescue effects not only through antioxidant activities but also via a combined ability to antagonize amyloid aggregation, suppress neuroinflammation, modulate signaling pathways, and decrease mitochondrial dysfunction. From the extensive *in vitro* and *in vivo* experimental evidence reviewed, RWP undoubtedly have strong potential to alleviate and/or attenuate the neurodegenerative process in AD and PD, making them ideal candidates for counteracting the multifaceted nature of these conditions. Yet, we have to be cautious in extrapolating findings from *in vitro* studies to the *in vivo* situation, since much of the existing *in vitro* data have utilized non-physiological concentrations of RWP and used the original molecule (aglycone) instead of the *in vivo* metabolites produced upon digestion and/or metabolic processing. Indeed, much of the recent data have consistently shown that the biological activities of metabolites may differ from the parent compound. At the same time, in order to efficiently translate experimental insights on RWP into clinical therapeutic benefit, it is essential to better characterize their metabolism, absorption profiles, and factors that influence bioavailability (179). Metabolism of natural compounds, such as RWP may be problematic in the clinical setting since they are metabolized by the same enzymes (e.g., cytochrome P450 enzymes and glucuronosyltransferases) that also metabolize clinically important drugs, such as warfarin and digoxin. In this manner, natural polyphenols may significantly alter the pharmacokinetic and pharmacodynamic properties of administered drugs, potentially increasing risk of toxicity (180, 181). To circumvent the drawback of poor biodisponibility, new delivery systems, such as the encapsulation of bioactive RWP in lipid nanocapsules appear to provide a promising frontier that could pave the way for the development of brain-targeted nutraceutical products (182). However, administration of nanoparticle preparations for prolonged periods may give rise to toxicity of the carriers with which the active compounds are complexed (181). Overall, resveratrol is the RWP most intensely studied to date. In the future, more attention should be paid to other phenolic components in grape or wine, including exploring the effect of novel combinations in formulations containing red wine phenols (e.g., GSE, Bioactive Dietary Polyphenol Preparation) for synergistic neuroprotective effects. Lastly, intervention studies will be required to utilize better-characterized disease biomarkers and more rigorous clinical outcomes. Ultimately, only the success of the clinical research will determine the relevance of RWP to be incorporated as key components in clinical practice or dietary guidelines to modulate the onset and/or progression of AD and PD.

## Author Contributions

All authors (MC, RC, and NV) co-wrote the manuscript. All authors read and commented on the manuscript.

## Conflict of Interest Statement

The authors declare that the research was conducted in the absence of any commercial or financial relationships that could be construed as a potential conflict of interest.

## Abbreviations

6-OHDA, 6-hydroxydopamine; αS, alpha-synuclein; Aβ, amyloid-beta; AD, Alzheimer’s disease; Akt, protein kinase B (PKB); ARE, antioxidant response element; BBB, blood–brain barrier; ERK1/2, extracellular signal-regulated protein kinase; GSE/GSPE, grape seed extract/grape seed polyphenolic extract; JNK, stress activated c-Jun N-terminal kinase; MAPK, mitogen-activated protein kinase; MCI, mild cognitive impairment; MeDi, Mediterranean diet; NF-κB, nuclear factor kappa B; Nrf2, nuclear factor erythroid 2-related factor 2; OS, oxidative stress; PD, Parkinson’s disease; PI3K, phosphoinositide 3-kinase; ROS, reactive oxygen species; RWP, red wine polyphenols.
